# Letter to the editor: Autochthonous transmission patterns of dengue virus serotype 2 in Italy: evidence from outbreaks in 2024

**DOI:** 10.2807/1560-7917.ES.2026.31.34.2600706

**Published:** 2026-08-27

**Authors:** Maíra Aguiar, Giulio Pisaneschi, Piero Manfredi, Alberto Landi, Nico Stollenwerk

**Affiliations:** 1 BCAM - Basque Center for Applied Mathematics, Bilbao, Spain; 2 Ikerbasque, Basque Foundation for Science, Bilbao, Spain; 3 University of Pisa, Pisa, Italy; 4 Institute of Clinical Physiology, National Research Council, Pisa, Italy

**Keywords:** dengue, reproduction number, stochastic transmission, epidemic threshold, modelling

**To the editor:** Molina Grané and colleagues provide an important reconstruction of the 2024 autochthonous dengue virus serotype 2 outbreaks in Italy and quantify transmission patterns across affected provinces [1]. Their analysis builds on the initial description of the large Fano outbreak and the public health measures implemented during its containment [2]. In Fano, they estimate a peak time-varying reproduction number (R_t_) > 3, comparable to values reported in endemic settings, followed by a decline below one after outbreak detection [1]. These findings raise an important question for dengue emergence in temperate Europe: does a large, realised outbreak with R_t_ > 1 necessarily identify the underlying transmission regime, or can similar patterns arise through stochastic amplification near the epidemic threshold?

An R_t_ > 1 indicates that transmission was increasing during the interval considered. However, even when such growth persists over several transmission generations, it does not necessarily establish that the underlying transmission system is intrinsically supercritical. Near the epidemic threshold, stochastic amplification can produce sizeable outbreaks under conditions for which transmission remains subcritical on average [3-6]. This distinction is especially important for mosquito-borne infections in temperate, non-endemic settings, where vector abundance and transmission potential vary sharply over short seasonal windows and small spatial scales [3-5].

For Fano, the key question is whether the observed epidemic trajectory uniquely identifies the underlying transmission regime. A large outbreak with R_t_ substantially above one may arise because transmission is genuinely supercritical, because conditions become transiently supercritical during a favourable seasonal window, or because stochastic amplification occurs in a system that is subcritical on average but close to the epidemic threshold. The smaller outbreaks in Modena and Chieti illustrate this uncertainty particularly well. In Modena, R_t_ hovered around one and subsequently declined well before outbreak detection, while in Chieti it exceeded one only briefly before falling again [1].

The patterns observed in Modena and Chieti make the near-threshold possibility particularly relevant. Near the epidemic threshold, stochastic mosquito-human transmission models can produce very different outcomes under similar average conditions: most introductions generate little or no onward transmission, while a minority produce apparently sustained growth over several transmission generations before declining [3,4,6]. A near-threshold interpretation therefore emphasises an underlying probability distribution of outcomes: many failed introductions, occasional focal clusters and rare but sizeable outbreaks under otherwise similar conditions [3-6]. The Italian outbreak reconstructions necessarily describe transmission chains that were detected. The authors themselves acknowledge that asymptomatic or mildly symptomatic infections may have gone unrecognised, that epidemiological links between some transmission foci cannot be excluded, and that genomic data would help determine whether the outbreaks resulted from single or multiple introductions [1]. Consequently, a realised outbreak alone cannot describe the full distribution of outcomes following virus introduction.

This uncertainty in the underlying transmission regime is also relevant when interpreting intervention effects. A decline in R_t_ following control measures is consistent with intervention effectiveness, but changes in transmission are not expected to be reflected instantaneously in an R_t_ trajectory reconstructed from symptom onset data. In Fano, municipality-wide adulticide interventions were implemented several days after outbreak detection [1]. The subsequent decline may therefore reflect the combined effects of seasonal reductions in vectorial capacity, control measures and stochastic fade-out. Disentangling these temporally overlapping mechanisms is necessary to quantify the specific contribution of interventions.

We therefore do not question the evidence for substantial local transmission during the Fano outbreak, as carefully characterised by Molina Grané et al., nor the high R_t_ estimated during its early phase. Rather, in view of the current dengue situation in Europe, we question whether these observations alone are sufficient to identify the underlying transmission regime as supercritical. Distinguishing genuine supercriticality from transient seasonal supercriticality and stochastic amplification near the epidemic threshold would require evidence across multiple virus introductions and transmission seasons, together with explicit comparison of these alternative mechanisms. The 2024 Italian outbreaks are consistent with Europe moving through a near-critical transition in which stochastic fluctuations can generate sizeable outbreaks before robust, recurrent supercritical transmission is established. Preparedness should therefore combine rapid response with probabilistic assessment of when introductions are most likely to amplify.
